# Cardiac Autonomic Modulation Is Different in Terms of Clinical Variant of Multiple Sclerosis

**DOI:** 10.3390/jcm9103176

**Published:** 2020-09-30

**Authors:** Monika Zawadka-Kunikowska, Łukasz Rzepiński, Julia L. Newton, Paweł Zalewski, Joanna Słomko

**Affiliations:** 1 Department of Hygiene, Epidemiology, Ergonomy and Postgraduate Education, Ludwik Rydygier Collegium Medicum in Bydgoszcz Nicolaus Copernicus University in Torun, M. Sklodowskiej-Curie 9, 85-094 Bydgoszcz, Poland; p.zalewski@cm.umk.pl (P.Z.); jslomko@cm.umk.pl (J.S.); 2 Department of Neurology, 10th Military Research Hospital and Polyclinic, 85-681 Bydgoszcz, Poland; luk.rzepinski@gmail.com; 3 Population Health Science Institute, The Medical School, Newcastle University, Framlington Place, Newcastle-upon-Tyne NE2 4HH, UK; Julia.Newton@newcastle.ac.uk

**Keywords:** multiple sclerosis, clinical variant, heart rate variability, blood pressure variability, sympathovagal ratio, cardiac autonomic imbalance

## Abstract

This study evaluates whether the cardiac autonomic response to head-up tilt test (HUTT) differs between patients with relapsing-remitting multiple sclerosis (RRMS) and those with progressive MS (PMS) as compared to healthy controls (HC). Baroreflex sensitivity, cardiac parameters, heart rate (HRV) and blood pressure variability (BPV) were compared between 28 RRMS, 21PMS and 25 HC during HUTT. At rest, PMS patients had higher values of the sympathovagal ratio, a low-frequency band HRV (LFnu-RRI) and lower values of parasympathetic parameters (HFnu-RRI, HF-RRI) compared to RRMS and HC. Resting values of cardiac parameters were significantly lower in RRMS compared to PMS patients. No intergroup differences were observed for post-tilt cardiac and autonomic parameters, except for delta HF-RRI with lower values in the PMS group. The MS variant corrected for age, sex and Expanded Disability Status Scale (EDSS) score was an independent predictor of changes in the sympathovagal ratio as measured by HRV. Furthermore, a higher overall EDDS score was related to a higher sympathovagal ratio, lower parasympathetic parameters at rest, and decrease post-tilt changes of the sympathovagal ratio of sBP BPV. Autonomic imbalance is markedly altered in the MS patient group compared to control changes were most pronounced in the progressive variant of MS disease. The MS variant appeared to have a potential influence on cardiac autonomic imbalance at rest.

## 1. Introduction

Multiple sclerosis (MS) is a chronic disorder characterized by autoimmune inflammation coupled to demyelination and followed later by central nervous system (CNS) neurodegeneration [1]. The differentiation between the relapsing-remitting phase (determined by partial or complete recovery) and the progressive phase, which is associated with steady progression in neurologic disability, can be challenging. Some studies indicate that up to 50% of relapsing-remitting MS (RRMS) patients will experience conversion to secondary progressive MS (SPMS) on average between 19 and 25 years after onset of the disease [2,3]. In addition, from onset, the course of primary progressive MS (PPMS) is associated with a worse prognosis than RRMS and SPMS [4,5].

Although reports related to the disease course in patients with MS are increasing, the nature of autonomic dysfunction (AD) seen frequently in MS remains unclear. It is well established that autonomic nervous system (ANS) impairment contributes to long-term disability in MS patients, but it is still not clear whether autonomic dysfunction results from damage to the central autonomic network (CAN) or whether additional immune-mediated peripheral, pathophysiology [6,7,8]. The interrelationship between AD and the clinical features of MS has been documented in several studies [9,10,11]. For example, disease activity in clinical relapses seems to be associated with sympathetic dysfunction, whereas disease progression could be linked with parasympathetic nervous system dysfunction, particularly in advanced stages of the disease [8]. Others suggest that sympathetic dysfunction is closely related to progression in clinical disability and autonomic imbalance in the progressive phenotype of MS [10]. Autonomic dysfunction induced by MS involves sympathovagal imbalance which leads to a higher incidence of cardiovascular disease morbidity [12,13]. A meta-analysis from 2015 reported the prevalence of cardiac AD as either 42% or 19%, depending on whether one or at least two abnormal autonomic test results were used to define AD [6]. Some reports have indicated that cardiac AD is altered more in patients with progressive MS than in relapsing-remitting RR [9,10,14]. Cardiac AD may include baroreflex dysfunction with orthostatic hypotension (OH), postural tachycardia syndrome (POTS), or decreased heart rate (HRV) and blood pressure variability (BPV) which is associated with reduced life expectancy [15]. Hence, it is important to know if cardiovascular AD is subtle or clinically relevant. Spectral analysis of beat-to-beat BPV and HRV are increasingly recognized as sensitive tools of cardiovascular autonomic regulation in patients with neurological diseases [16].

We hypothesized that imbalance in sympathetic and parasympathetic cardiac modulation may be related to clinical features of multiple sclerosis (MS), in particular disease disability (Expanded Disability Status Scale (EDSS) score), disease duration and a clinical variant of the disease. This study evaluates whether cardiac autonomic response to head-up tilt test (HUTT) differs between patients with relapsing-remitting (RRMS) and those with progressive MS (PMS) as compared to healthy controls (HC).

## 2. Experimental Section

### 2.1. Participants

Forty-nine patients with MS and 25 age-matched healthy controls (HC) were compared in this study. Data collection was conducted between 2017 and 2019. Depending on the time the diagnosis was established, the McDonald’s criteria and Polman et al.’s criteria were applied [17,18]. The clinical course of MS was determined in accordance with the Lublin and Reingold consensus [19]. The MS patients were divided into two groups: relapsing-remitting (RRMS) and progressive (PMS), with the PMS group including patients with PPMS and SPMS. The patients’ disability was evaluated according to the Kurtzke Expanded Disability Status Scale (EDSS) [20]. Using the EDSS, patients with mild (EDSS ≤ 3.5), moderate (4.0 ≤ EDSS ≤ 5.5) and severe disability (EDSS ≥ 6.0) were distinguished. EDSS is the most widely used scale to describe the disability as well as disease progression of MS patients. EDSS scores range from 0 to 10 in 0.5 step intervals, with 0 indicating no disability, and 10 denoting death from MS. At a mild disability level, the EDSS score is determined by neurological examination and includes only subjects with unrestricted ambulation. Patients with a moderate disability level usually have some distance limitation but without aid and those with severe disability always require walking assistance [20,21].

The inclusion criteria for patients with MS were: confirmed diagnosis of MS, EDSS score less than 7 and no clinical relapses within 90 days prior to the study. Subjects with any other diseases affecting autonomic function including ischemic heart disease, hypertension, hypothyroidism, hyperthyroidism, diabetes mellitus and treatment with beta-blockers, anticholinergic or antiarrhythmic were excluded from the study. Controls were recruited from the local community of Bydgoszcz, Poland. Controls who manifested central or peripheral nervous system lesions and any other disease known to affect the autonomic nervous system were excluded.

The study protocol was approved by the Bioethical Committee of Collegium Medicum in Bydgoszcz, Nicolaus Copernicus University in Torun (KB 747/2017). All participants participated voluntarily and gave their written informed consent according to the declaration of Helsinki.

### 2.2. Cardiac and Autonomic Measures

All measurements were performed under standardized conditions meeting criteria for functional testing of the ANS, between 08:00 a.m. and 12:00 p.m [22,23]. The study room was quiet and darkened, and the air conditioning system maintained a stable temperature (22 ± 1 °C) and air humidity. Subjects were asked to refrain from drinking coffee, smoking, alcohol and exercise for at least 12 h prior to the study. All measurements were assessed in a supine position for 15 min and during the head-up tilt test, using a 70° angle of tilt for 5 min. Cardiac and autonomic measurements were calculated from data acquired noninvasively with a Task Force Monitor System (TFM, CNSystems, Medizintechnik, Graz, Austria).

The heart rate (HR) was measured with an electrocardiogram (ECG), while beat-to-beat systolic (sBP) and diastolic blood pressure (dBP) were measured in the right arm by a vascular unloading technique that was compared automatically to the oscillometric blood pressure measured on the contralateral arm [24]. The TFM software evaluated power spectral analysis for heart rate variability (HRV) and blood pressure variability (BPV) via the adaptive autoregressive model (AAR) proposed by Bianchi et al. [25] using a recursive least-squares algorithm [26]. All functions of the TFM were validated and successfully used in a number of clinical studies [24,25,27]. HRV and BPV have become substantial diagnostic tools for the detection of cardiovascular autonomic regulation in neurological diseases [7,10,16,25,28].

TFM calculates total power spectral density (PSD) and three main frequency bands: very-low-frequency (VLF), low-frequency (LF) and high-frequency (HF); however, only two of these were considered as there were short-term autonomic regulations of beat-to-beat HR and BP signals. The LF band (LF 0.05–015 Hz) and HF band (HF 0.15–0.4 Hz) were calculated in both absolute values and normalized units (LFnu-RRI, HFnu-RRI for heart rate variability and LFnu-sBP, HFnu-sBP, LFnu-dBP and HFnu-dBP for systolic and diastolic blood pressure variability) [29]. Keeping in mind the limitations of spectral analysis in quantifying autonomic nervous system tone by power spectral densities, the LF band reflected the combined sympathetic and parasympathetic modulation of the sinoatrial (SA) node and vasomotor function, while the HF band referred to parasympathetic modulation of cardiac activity. Frequency-domain parameters, such as PSD, LF and HF, were considered to be reliable markers of autonomic regulation. The ratio between LF and HF bands (LF/HF ratio) for HRV and BPV represented the sympathetic–parasympathetic balance [29]. Baroreceptor sensitivity (BRS) was calculated using the spontaneous sequence method as the slope of the linear regression between beat-to-beat sBP values (mmHg) [27].

Short-term HRV analyses have different advantages and disadvantages. The advantages of short-term HRV analysis include: dynamic HRV change within a short period; shorter time for data processing compared to long-term analysis; and convenience in controlling the confounding factors such as body position, physical activity and respiration. Short-term frequency HRV analysis may not be stable due to the constant fluctuation of recordable signals [29,30]. In our study, HRV and BPV data were exported from the Task Force Monitor program into Microsoft Excel for further analysis. All data were then imported into Statistica 13. The AAR model may produce outliers when analyzing RR intervals, thus all HR beat-to-beat data were filtered using Grubbs’s test for outliers’ elimination. This method of filtering is well-documented and has a strong mathematical background) [31].

The diagnosis of POTS was made if there was an increase in HR during a maximum of 10 min of upright tilt of at least 30 beats per minute (bpm), in the absence of either classical or delayed orthostatic hypotension. Orthostatic hypotension (OH) was defined as a drop in blood pressure (BP) of at least 20 mm Hg for sBP or 10 mm Hg for dBP within 3 min during a head-up tilt test [32].

### 2.3. Statistical Analysis

All data are presented as mean ± SD. The normal distribution of the study variables was verified with the Shapiro-Wilk test. Differences in the distribution of qualitative variables were determined with the Χ^2^-test, while the differences in quantitative variables were determined with the use of a parametric t-test or a nonparametric Mann-Whitney test. Multiple comparisons were performed by analysis of variance, followed by Tukey’s HSD test or by the Kruskal-Wallis rank-sum test. The strength and significance of the correlation between selected variables were calculated using the nonparametric Spearman’s test. The multiple regression model, based on four predictors (age, sex, EDSS and MS variant (RRMS or PMS)), was also used in order to determine significant predictors for HRV and BPV variables. The level of significance for all tests was set at *p* < 0.05.

## 3. Results

In the group of MS patients, the mean age was 46.3 ± 10.47 years (range: 23–67) and 79.6% were female (39 female, 10 male). Patient disability evaluated on the EDSS scale ranged from 0.5 to 7 points patient disability status which indicates mild disease for the majority of patients (53.6%), through moderate (28.6%) to severe (18.4%). Out of 49 MS patients, relapsing-remitting (RRMS) and progressive variants (PPMS and SPMS) of the disease were observed in 59.2% and 40.8% (6.1% and 34.7%) patients, respectively. Patients in the RRMS group compared to the PMS group were significantly younger (41.3 ± 10 vs. 52.9 ± 8.1, *p* < 0.001), had a shorter disease duration (8.3 ± 6.7 vs. 12.5 ± 7.4, *p* < 0.043) and lower EDSS values (2.3 ± 1.5 vs. 5.0 ± 1.0, *p* < 0.001), respectively. There were significant sex differences between the RRMS (1 male, 27 female) and PMS groups (9 male, 12 female), respectively. A total of 13 patients (26.5%) received immunomodulatory drugs (IMDs): eight interferon-beta, four glatiramer acetate and one natalizumab. Among the MS patients, the autonomic symptoms were most commonly manifested as orthostatic disorders (65.3%), followed by vertigo (60.2%), pupillomotor disorders (53.1%), sleep disorders (46.9%) and urinary bladder dysfunctions (42.8%). MS patients had a significantly higher frequency of orthostatic disorders, vertigo, thermoregulatory disorders, episodes of stomach ache, postmeal symptoms, diarrhea, urinary bladder dysfunctions, sexual dysfunctions, sleep disorders and pupillary disorders, as compared to the control group, *p* < 0.05 (Table 1).

### 3.1. Cardiac and Autonomic Assessment: Comparisons MS and Control Group

The majority (96%) of MS subjects had normal heart and blood pressure responses to standing. Only two MS patients (4.1%) had POTS and two (4.1%) OH (Table 2). At rest, the MS patients and controls had comparable values of HR and BP, and no significant differences in cardiac parameters were observed among the two groups (*p* > 0.05; Table 3). In addition, LFnu-RRI (*p* = 0.038), LF/HF-RRI (*p* = 0.029) and LF/HF ratio (*p* = 0.042) were found to be significantly higher in MS subjects compared to the control group. MS patients were characterized by significantly lower values associated with parasympathetic activity, i.e., HFnu-RRI (*p* = 0.04) and HFnu-dBP (*p* = 0.049). In contrast, no significant differences were observed between the groups in other sBPV, dBPV and BRS parameters (*p* > 0.05; Table 3). No intergroup differences were observed for post-tilt cardiac and ANS parameters, except for delta LF-sBP (*p* = 0.047) and delta HF-sBP (*p* = 0.04).

### 3.2. Cardiac and Autonomic Assessment: Comparison Clinical Variant of MS and Control Group

**Figure 1:**
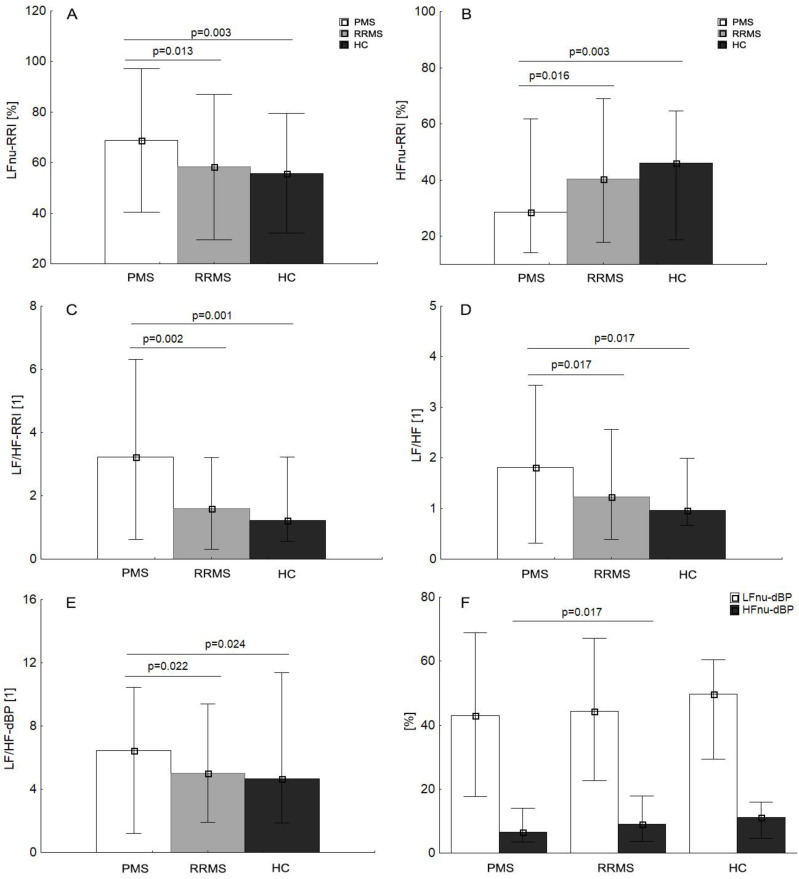
Multiple sclerosis (MS) subgroup (PMS, progressive MS; RRMS, relapsing-remitting MS) mean values (±SD) at rest of LFnu-RRI, low-frequency R-R interval in normalized units (**A**); HFnu-RRI, high-frequency R-R interval in normalized units (**B**); LF/HF-RRI, ratio between low and high band for heart rate variability (**C**); LF/HF, ratio between low and high band for heart rate and blood pressure variability (**D**); LF/HF-dBP, ratio between low and high band for diastolic blood pressure variability (**E**); LFnu-dBP, low frequency of diastolic blood pressure variability in normalized units; LFnu-dBP, low frequency of diastolic blood pressure variability in normalizes units (**F**); HFnu-dBP, high frequency of diastolic blood pressure variability in normalized units (**F**), respectively, compared to healthy controls (HC).

At rest, PMS patients were characterized by significantly higher LFnu-RRI (*p* = 0.013), sympathovagal balance ratio (LF/HF-RRI, LF/HF, LF/HF-dBP) and lower values of HFnu-RRI, compared to the RRMS and HC. (Figure 1A–E). Therefore, PMS showed significantly lower HFnu-dBP, as compared to HC (Figure 1F). RRMS patients as compared to PMS were characterized by significantly lower values of LFnu-sBP (*p* = 0.017) and cardiac parameters, i.e., HR (*p* = 0.044), dBP (*p* = 0.008), mBP (*p* = 0.045) and higher HF-RRI (*p* = 0.037; Table 4). In contrast, no significant differences were observed between the RRMS and control groups in cardiac, autonomic and BRS parameters (*p* > 0.05). An orthostatic response to the tilt test in the MS subgroups and HC group was similar and characterized by an HR and blood pressure increase with similar sympathetic reactivity, but without statistical significance (*p* > 0.05). No intergroup differences were observed for post-tilt cardiac and ANS parameters, except for delta HF-RRI (*p* = 0.033) with lower values in the PMS group.

### 3.3. Relationship between Cardiovascular and Autonomic Parameters, Disease Duration and EDSS Score

In MS patients, the EDDS score was positively correlated with values of age (*R* = 0.46; *p* = 0.002), HR (*R* = 0.37; *p* = 0.015), dBP (*R* = 0.47; *p* = 0.001), mBP (*R* = 0.38; *p* = 0.008), LF/HF-dBP (*R* = 0.30; *p* = 0.032), LF/HF-sBP (*R* = 0.31; *p* = 0.035) and post-tilt changes in delta HF-dBP (*R* = −0.34; *p* = 0.018). Furthermore, the EDSS score was negatively correlated with HFnu-sBP (*R* = −0.30; *p* = 0.01), HFnu-dBP (*R* = −0.34; *p* = 0.028) and delta LF/HF-sBP (*R* = −0.30; *p* = 0.029). The disease duration was positively associated with age (*R* = 0.45; *p* = 0.002). All significant correlations have been shown in Appendix A (Table A1 and Table A2). The multiple regression model statistically significantly predicts the LF/HF-RRI ratio (F = 4.42, *p* < 0.006), with an *R*^2^ = 0.28. MS variant (RRMS or PMS), corrected for age, sex and EDDS score, was a statistically significant predictor for the presence of autonomic balance measured with LF/HF ratio (β = 0.51, *p* = 0.021). Age, sex and EDSS score were not identified as independent predictors for the presence of AD measured with the LF/HF-RRI ratio. Cardiac parameters (dBP, mBP) were predominantly predicted by sex variable, whereas age was a significant predictor for HF-RRI (β = −0.34, *p* = 0.041). Significant predictors for cardiac, HRV, BPV parameters for MS group are presented in Table 5.

## 4. Discussion

Our study investigated cardiovascular autonomic modulation using short-term spectral (HRV and BPV) analysis in patients with different MS phenotypes compared to age-matched healthy subjects. The main finding of this study is that the MS variant (RRMS or PMS) corrected for age, sex, EDSS score, is an independent predictor of changes in sympathovagal ratio as measured with HRV. Furthermore, a higher overall EDDS score was related to a higher sympathovagal ratio (LF/HF-sBP, LF-HF-dBP), and lower parasympathetic parameters (HFnu-dBP, HFnu-sBP) at rest, and decreased post-tilt changes of LF/HF-sBP.

Our study has confirmed previous findings, which indicate that imbalance in the autonomic nervous system is a common feature of MS patients [9,10,33], with a significant difference in patterns of dysautonomia in patients with RRMS and PPMS. Consistent with the previous study, we found that PMS patients had significantly higher values of the sympathetic–parasympathetic ratio, LFnu-RRI, reflecting a shift of the sympathovagal modulation toward sympathetic predominance, as compared to the RRMS and HC. In addition, lower values of parasympathetic parameters (Hfnu-RRI, HF-RRI) in PMS may suggest lower cardiac parasympathetic modulation to the sinoatrial node compared to RRMS [9]. Some studies indicate lower overall HRV parameters in MS (progressive and RRMS [34] or RRMS alone [7,35,36,37] than in HC. Others found higher LF in progressive MS than in RRMS and HC [10]. This discrepancy may be explained by differences in the number of RRMS and PMS patients, level of disability and disease duration. In response to head-up tilt, both MS and control groups demonstrated an increase in cardiac sympathetic modulation, whereas the decrease in HF-post-tilt changes was lower in PMS, indicating slightly an impairment of sympathetic function or insufficient withdrawal of cardiac parasympathetic modulation. LFnu-RRI was slightly, but not significantly lower in MS compared to HC in all experimental conditions.

Furthermore, a higher EDDS score was related to a higher sympathovagal ratio (LF/HF-sBP, LF-HF-dBP), lower parasympathetic parameters (HFnu-dBP, HFnu-sBP) of BPV at rest, and decrease post-tilt changes of LF/HF-sBP. This may reflect progressive concomitant alteration of both cardiac sympathetic and parasympathetic interplay with a decreased sympathetic modulation [9,10,38,39]. Interestingly, orthostatic intolerance (POTS and OH) were found in both MS groups which may indicate a more active disease course associated with sympathetic nervous system dysfunction [10,40]. Recent studies confirm that MS lesions are centered around CAN structures such as the corpus callosum, peri- and paraventricular structures [41] and nuclei or pathways that modulate baroreflex sensitivity and cardiovascular autonomic function [42]. The involvement of the central autonomic network in autonomic cardiovascular modulation is also supported by the study of Winder et al. who showed associations between a shift of cardiovascular sympathetic–parasympathetic balance toward increased sympathetic modulation and left insular and hippocampal lesions [43].

We did not find significant correlation between the disease duration and changes in cardiovascular autonomic parameters in the MS group which is consistent with previous studies [7].

Potential mechanisms underlying the sympathetic overactivity observed in PMS patients may reflect the bidirectional relationship between the immune system and the ANS [44]. Previous studies showed that the influence of the sympathetic nervous system (SNS) on the immune response depends largely on the time point of sympathetic activation. A decreased SNS tone promoted proinflammatory effects, whereas a increased SNS tone resulted in an anti-inflammatory response during the chronic phase of inflammation [45,46]. MS may affect the ANS not only via the neurodegeneration process, but also by modulating the SNS peripherally through catecholamine release from inflammatory lesions, or by inflammatory induced expression of β-adrenergic receptors [6]. Namely, differences in the sympathetic–parasympathetic ratio in MS phenotypes may be explained by the fact that immune stimulation from chronic inflammation causes a maladaptive disease-inducing and consolidating sympathetic response in an attempt to maintain allostasis [47]. Therefore, it is likely that altered autonomic imbalance may contribute to the pathogenesis of MS or could be common a consequence of the disease itself [8]. We did not find a significant difference in autonomic parameters between the RRMS and HC groups. This is in line with the study by Studer et al. who found that in RRMS patients, disease activity, even subclinical, was associated with lower rest LFnu, whereas stable RRMS patients did not differ from healthy controls. Moreover, sympathetic reactivity can be related to plastic reserves in RRMS because patients with higher sympathetic reactivity did not show any clinical signs of ongoing brain inflammation [10] Chronic neuroinflammation may also promote the development of reduced central parasympathetic together with a suppressive role for the SNS in the CNS immune response [44,47] The PMS group had higher values of HFnu-dBP compared to HC; however, the age score was a significant predictor of changes for HFnu-dBP. These results suggest that proper age-matching is needed to differentiate between disease-related pathophysiology and normal aging [48].

In several chronic immune-mediated inflammatory diseases such as rheumatoid arthritis, systemic lupus erythematosus, inflammatory bowel disease and SjÖgren’s syndrome, the tone of the sympathetic nervous system (SNS) is also increased. Systemic inflammation and immune dysfunction increase morbidity and mortality by affecting multiple organ systems, especially the heart and kidney [47]. Along these lines, PMS patients have significantly higher cardiac variables compared to RRMS which suggest the higher sympathetic modulation of HR and dBP. Another possible explanation for increased cardiac values is the upregulation of b-adrenergic receptors on peripheral blood mononuclear cells [40]. Therefore, prolonged stimulation of β-AR receptors in the myocardium can drive cardiomyocyte hypertrophy, mitogenesis of cardiac fibroblasts, and the development of heart failure [49,50]. Similarly, subclinical cardiac involvement among 40 active RRMS patients was also confirmed by Olindo et al. [51], who found a reduced left and right ventricular ejection fraction, as compared to controls.

There are several limitations to this study. First, the sample size is relatively small. In our study, statistical power appears to be generally, which warrants further investigation. Future research considering case-control differences in HRV and BPV should include effect size distributions to convey group difference magnitudes [52]. Second, patients and controls were not matched for age and gender, which could independently affect the acquisition of autonomic dysfunction. HRV can also be significantly affected, directly or indirectly, by various groups of drugs. Therefore, the use of medications in subjects should be adequately assessed when interpreting HRV indexes [53]. Our study did not evaluate the impact of the use of IMDs for the management of cardiovascular risk in patients with MS phenotypes. None of the patients did not take fingolimod used to treat RRMS. Fingolimod is known to reduce cardiac autonomic modulation (HR reduction) and baroreflex sensitivity at rest, as well as to diminish cardiovagal responses to autonomic challenges [54] Finally, researches should consider using machine learning approaches for clinical diagnosis based on physiological cardiac data [55].

## 5. Conclusions

We conclude that autonomic imbalance is markedly altered in the MS patient group compared to controls, changes were most pronounced in the progressive variant of MS disease. The MS variant appeared to have a potential influence on cardiac autonomic imbalance at rest. Furthermore, higher overall EDDS score was related to a higher sympathovagal ratio, lower parasympathetic parameters at rest, and decrease post-tilt changes of the sympathovagal ratio of systolic blood pressure variability. Our results indicate the need for assessment of cardiovascular autonomic function, especially heart and blood pressure response to orthostatic stress when developing therapies aimed at improving functional mobility. Future research should identify objective markers of autonomic dysfunction in the large MS population including patients in early and advanced stages of MS disease.

## Figures and Tables

**Table 1:** Subjects characteristics.

	MS Patients	HC	*p*-Value
Number of subjects	49	25	
Age (years)	46.0 ± 11.0	42.3 ± 12.4	0.164
Sex (male/female)	10/39	5/20	0.967
MS variant, n (%)			
RRMS	28 (57.1%)		
SPMS	17 (34.7%)		
PPMS	4 (6.1%)		
Disease duration (years), mean (range)	10.1 ± 7.2 (0.5–28)		
EDSS score	3.5 ± 1.0 (0.5–7)		
Mild	26 (53.6%)		
Moderate	14 (28.6%)		
Severe	9 (18.4%)		
Localization of the First Demyelinating Lesions, n (%)			
Supratentorial and optic nerves	32 (65.3%)		
Spinal cord	12 (24.5%)		
Cerebellum	4 (8.2%)		
Brain stem	1 (2.0%)		
Autonomic symptoms, n (%)			
Orthostatic disorders	32 (65.3%)	3 (12.0%)	<0.001
Vertigo	30 (61.2%)	1 (4.0%)	<0.001
Arrhythmia	13 (26.5%)	3 (12.0%)	0.150
Vasomotor disorders	8 (17.0%)	4 (16%)	0.911
Secretory disorders	10 (20.4%)	1 (4.0%)	0.060
Thermoregulatory disorders	17 (34.7%)	1 (4.0%)	0.036
Stomach ache	17 (34.6%)	1 (4.0%)	0.047
Constipation	8 (16.3%)	4 (16.0%)	0.971
Diarrhea	21 (42.9%)	1 (4%)	<0.001
Postmeal symptoms	7 (14.3%)	1 (4%)	0.047
Urinary bladder dysfunctions	21 (42.9%)	1 (4%)	<0.001
Sexual dysfunction	15 (30.6%)	0 (0%)	0.008
Sleep disorders	23 (46.9%)	5 (20.0%)	0.023
Pupillary disorders	26 (53.1%)	1 (4%)	<0.001

Multiple sclerosis (MS), healthy controls (HC), relapsing-remitting (RRMS), secondary progressive MS (SPMS), primary progressive MS (PPMS), Expanded Disability Status Scale (EDSS).

**Table 2:** Cardiac autonomic tests: comparisons multiple sclerosis (MS) and control group.

Group	MS	HC
Cardiac autonomic tests	*n* (%)	*n* (%)
Blood pressure response to standing (fall in BP in mmHg)		
Normal	47 (96%)	25 (100%)
Abnormal (OH)	2 (4%)	0 (0%)
Heart rate response to standing (increase in HR in bpm/min)		
Normal	47 (96%)	25 (100%)
Abnormal (POTS)	2 (4%)	0 (0%)

Multiple sclerosis (MS), healthy controls (HC), orthostatic hypotension (OH), postural tachycardia syndrome (POTS).

**Table 3:** Mean ± SD of resting and during tilt test cardiac autonomic measures for patients with MS and healthy controls (HC).

Group	MS	HC	MS	HC
	**Baseline**		**Delta (change baseline-tilt)**	
Cardiac data				
HR (1/min)	66.5 ± 7.3	66.3 ± 9.2	12.7 ± 7.7	14.2 ± 8.1
sBP (mmHg)	1134.0 ± 12.0	115.0 ± 11.4	12.1 ± 14.2	11.7 ± 9.9
dBP (mmHg)	73.4 ± 8.9	75.3 ± 8.5	16.1 ± 11.5	14.6 ± 8.0
mBP (mmHg)	90.4 ± 9.8	92.5 ± 9.2	16.1 ± 16.8	13.1 ± 8.5
Heart rate variability (HRV)				
LFnu-RRI (ms^2^)	62.8 ± 15.1	55.7 ± 11.8 *	12.1 ± 15.0	13.7 ± 17.0
HFnu-RRI (ms^2^)	37.3 ± 15.0	44.3. ± 11.8 *	−12.2 ± 14.9	−13.7 ± 17.0
LF-RRI (ms^2^)	746.6 ± 896.0	619.7 ± 514.9	−281.4 ± 895.2	−44.7 ± 498.0
HF-RRI (ms^2^)	507.9 ± 703.0	575.3 ± 668.5	−381.5 ± 659.5	−342.4 ± 550.4
PSD-RRI (ms^2^)	1713.8 ± 1700.8	1538.3 ± 1262.5	−955.8 ± 1573.8	−432.3 ± 1024.6
LF/HF-RRI [1]	2.4 ± 1.5	1.6 ± 0.9 *	2.4 ± 3.0	1.8 ± 2.5
LF/HF [1]	1.5 ± 0.9	1.3 ± 0.7	1.6 ± 2.0	1.5 ± 1.5
Systolic and diastolic pressure variability (BPV)				
LFnu-dBP (%)	43.7 ± 12.2	48.5 ± 11.9	6.9 ± 8.8	3.5 ± 11.8
HFnu-dBP (%)	9.1 ± 3.9	11.1 ± 4.7 *	0.8 ± 2.3	0.4 ± 3.4
LF-dBP (%)	3.9 ± 2.6	4.1 ± 3.5	−0.4 ± 1.0	−0.9 ± 1.7
HF-dBP (%)	0.8 ± 0.7	0.9 ± 0.8	−0.2 ± 0.3	−0.2 ± 0.3
PSD-dBP (mmHg^2^)	8.8 ± 5.2	8.2 ± 5.9	−2.0 ± 1.8	−1.6 ± 2.7
LF/HF-dBP	5.7 ± 2.6	5.2 ± 2.5	0.4 ± 2.0	0.6 ± 2.6
LF/HF	1.5 ± 0.9	1.3 ± 0.7 *	1.6 ± 2.0	1.2 ± 1.5
LFnu-sBP (%)	43.2 ± 12.1	42.2 ± 12.0	9.0 ± 11.6	5.9 ± 11.0
HFnu-sBP (%)	11.5 ± 6.8	12.6 ± 6.8	3.2 ± 4.9	2.2 ± 4.4
LF-sBP (%)	6.0 ± 5.6	6.3 ± 6.2	−0.4 ± 2.1	−1.5 ± 2.7 *
HF-sBP (%)	1.4 ± 1.0	1.8 ± 1.9	−0.1 ± 0.5	−0.4 ± 0.7 *
PSD-sBP (mmHg^2^)	135 ± 9.5	14.0 ± 11.2	−3.3 ± 3.5	−4.1 ± 4.9
LF/HF-sBP [1]	4.8 ± 2.6	4.1 ± 1.7	−0.1 ± 1.7	0.1 ± 1.5
LF/HF [1]	1.5 ± 0.9	1.1 ± 0.7	1.7 ± 2.1	1.2 ± 1.5
BRS (ms/mmHg)	60.0 ± 21.5	69.3 ± 19.3	-	-
Total BEI (%)	15.8 ± 11.4	18.7 ± 10.2	-	-

MS, multiple sclerosis; HC, healthy controls; HR, heart rate; sBP, systolic blood pressure; dBP, diastolic blood pressure; mBP, mean blood pressure; LFnu-RRI, low-frequency R-R interval in normalized units; HFnu-RRI, high frequency R-R interval in normalized units; LF-RRI, low-frequency R-R interval; HF-RRI, high-frequency R-R interval; PSD-RRI, power spectral density R-R interval; LF/HF, ratio between low and high band for heart rate and blood pressure variability; LF/HF-RRI, ratio between low and high band for heart rate variability; LFnu-dBP, low frequency of diastolic blood pressure variability in normalized units; HFnu-dBP, high frequency of diastolic blood pressure variability in normalized units; LF-dBP, low frequency of diastolic blood pressure variability; HF-dBP, high frequency of diastolic blood pressure variability; PSD-dBP, power spectral density of diastolic blood pressure variability; LF/HF-dBP, ratio between low and high band for diastolic blood pressure variability; LFnu-sBP, low frequency of systolic blood pressure variability in normalized units; HFnu-sBP, high frequency of systolic blood pressure variability in normalized units; LF-sBP, low frequency of systolic blood pressure variability; HF-sBP, high frequency of systolic blood pressure variability; PSD-sBP, power spectral density of systolic blood pressure variability; LF/HF-sBP, ratio between low and high band for systolic blood pressure variability; BRS, baroreflex sensitivity; total BEI, baroreflex effectiveness; nu, normalized values; statistically significant differences are indicated with * *p* < 0.05.

**Table 4:** Mean ± SD of resting and during tilt test cardiac autonomic measures for patients with RRMS, PMS and HC.

Group	RRMS	PMS	HC	RRMS	PMS	HC
	Baseline			Delta (change baseline-tilt)		
Cardiac data						
HR (1/min)	64.3 ± 7.4	69.4 ± 6.2	66.3 ± 9.2	13.8 ± 8.1	11.2 ± 6.2	14.2 ± 8.1
sBP (mmHg)	110.6 ± 11.1	117.2 ± 12.5	115.0 ± 11.4	141 ± 9.9	9.5 ± 12.5	11.7 ± 9.9
dBP (mmHg)	70.0 ± 8.1	77.9 ± 8.0 *	75.3 ± 8.5	19.0 ± 8.0	12.4 ± 8.0	14.6 ± 8.0
mBP (mmHg)	87.3 ± 8.9	94.5 ± 9.7 *	92.5 ± 9.2	16.8 ± 8.5	15.2 ± 9.7	13.1 ± 8.5
Heart rate variability (HRV)						
LFnu-RRI (ms^2^)	58.3 ± 14.3	68.9 ± 14.2 *	55.7 ± 11.8	15.8 ± 17.0	7.1 ± 14.2	13.7 ± 17.0
HFnu-RRI (ms^2^)	41.7 ± 14.3	31.4 ± 14.1 *	44.3 ± 11.8	−15.8 ± 17.0	−7.4 ± 14.1	−13.7 ± 17.0
LF-RRI (ms^2^)	790.1 ± 645.3	688.5 ± 1165.5	619.7 ± 514.9	−363.1 ± 498.0	−172.4 ± 1165.5	−44.7 ± 498.0
HF-RRI (ms^2^)	675.1 ± 851.3	285.1 ± 341.3 *	575.3 ± 668.5	−544.7 ± 550.4	−163.9 ± 341.3 *	−342.4 ± 550.4
PSD-RRI (ms^2^)	2044.4 ± 1749.0	1273.0 ± 1566.9	1538.3 ± 1262.5	−1304.6 ± 1024.6	−490.8 ± 1566.9	−432.3 ± 1024.6
LF/HF-RRI [1]	1.8 ± 1.1	3.1 ± 1.7 *	1.6 ± 0.9	2.4 ± 2.5	2.5 ± 1.7	1.8 ± 2.5
LF/HF [1]	1.3 ± 0.7	1.8 ± 1.0 *	1.3 ± 0.7	1.5 ± 1.5	1.7 ± 1.0	1.5 ± 1.5
Systolic and diastolic pressure variability (BPV)						
LFnu-dBP (%)	44.3 ± 11.1	42.9 ± 13.8	48.5 ± 11.9	8.0 ± 11.8	5.5 ± 13.8	3.5 ± 11.8
HFnu-dBP (%)	10.2 ± 3.9	7.6 ± 3.5 *	11.1 ± 4.7	0.3 ± 3.4	1.4 ± 3.5	0.4 ± 3.4
LF-dBP (%)	3.8 ± 1.9	3.9 ± 3.4	4.1 ± 3.5	−0.4 ± 1.7	−0.4 ± 3.4	−0.9 ± 1.7
HF-dBP (%)	0.9 ± 0.8	0.6 ± 0.5	0.9 ± 0.8	−0.2 ± 0.3	−0.1 ± 0.5	−0.2 ± 0.3
PSD-dBP (mmHg^2^)	8.9 ± 4.8	8.6 ± 5.8	8.2 ± 5.9	−2.0 ± 2.7	−2.0 ± 5.8	−1.6 ± 2.7
LF/HF-dBP	5.1 ± 2.4	6.6 ± 2.7 *	5.2 ± 2.5	0.7 ± 2.6	−0.0 ± 2.7	0.6 ± 2.6
LF/HF	1.3 ± 0.7	1.8 ± 1.0 *	1.3 ± 0.7	1.5 ± 1.5	1.7 ± 1.0	1.2 ± 1.5
LFnu-sBP (%)	40.7 ± 10.3	46.5 ± 13.7 *	42.2 ± 12.0	11.5 ± 11.0	5.7 ± 13.7	5.9 ± 11.0
HFnu-sBP (%)	11.5 ± 5.4	11.5 ± 8.5	12.6 ± 6.8	2.8 ± 4.4	3.7 ± 8.5	2.2 ± 4.4
LF-sBP (%)	5.2 ± 2.6	7.2 ± 8.1	6.3 ± 6.2	−0.1 ± 2.7	−0.7 ± 8.1	−1.5 ± 2.7
HF-sBP (%)	1.4 ± 0.8	1.5 ± 1.3	1.8 ± 1.9	−0.1 ± 0.7	0.1 ± 1.3	−0.4 ± 0.7
PSD-sBP (mmHg^2^)	13.3 ± 7.40	13.9 ± 12.0	14.0 ± 11.2	−3.5 ± 4.9	−2.9 ± 12.0	−4.1 ± 4.9
LF/HF-sBP [1]	4.3 ± 2.0	5.5 ± 3.1	4.1 ± 1.7	0.4 ± 1.5	−0.7 ± 3.1	0.1 ± 1.5
LF/HF [1]	1.2 ± 0.6	2.0 ± 1.1	1.1 ± 0.7	1.7 ± 1.5	1.8 ± 1.1	1.2 ± 1.5
BRS (ms/mmHg)	58.9 ± 20.4	61.4 ± 23.5	69.3 ± 19.3	-	-	-
Total BEI (%)	18.5 ± 12.8	12.2 ± 7.9	18.7 ± 10.2	-	-	-

MS, multiple sclerosis; PMS, progressive MS; RRMS, relapsing-remitting MS; HC, healthy controls; HR, heart rate; sBP, systolic blood pressure; dBP, diastolic blood pressure; mBP mean blood pressure; LFnu-RRI, low-frequency R-R interval in normalized units; HFnu-RRI, high frequency R-R interval in normalized units; LF-RRI, low-frequency R-R interval; HF-RRI, high-frequency R-R interval; PSD-RRI, power spectral density R-R interval; LF/HF, ratio between low and high band for heart rate and blood pressure variability; LF/HF-RRI, ratio between low and high band for heart rate variability; LFnu-dBP, low frequency of diastolic blood pressure variability in normalized units; HFnu-dBP, high frequency of diastolic blood pressure variability in normalized units; LF-dBP, low frequency of diastolic blood pressure variability; HF-dBP, high frequency of diastolic blood pressure variability; PSD-dBP, power spectral density of diastolic blood pressure variability; LF/HF-dBP, ratio between low and high band for diastolic, blood pressure variability; LFnu-sBP, low frequency of systolic blood pressure variability in normalized units; HFnu-dBP, high frequency of systolic blood pressure variability in normalized units; LF-sBP, low frequency of systolic blood pressure variability; HF-sBP, high frequency of systolic blood pressure variability; PSD-sBP, power spectral density of systolic blood pressure variability; LF/HF-sBP, ratio between low and high band for systolic blood pressure variability; BRS, baroreflex sensitivity; total BEI, baroreflex effectiveness; nu, normalized values; statistically significant differences are indicated with * *p* < 0.05.

**Table 5:** Multivariate analysis e prediction of frequency domain and cardiac variables by clinical features.

Dependent Variables	Independent Variables	β	SE	t	*p*-Value
HR *R* = 0.45; *R*^2^ = 0.20 F(4.42) = 2.7; *p* < 0.042	Sex	0.02	0.16	0.15	0.880
	Variant	0.33	0.22	1.48	0.147
	Age	−0.28	0.16	−1.72	0.093
	EDSS	0.22	0.20	1.11	0.272
dBP *R* = 0.6; *R*^2^ = 0.36 F(4.42) = 16.67; *p* < 0.001	Sex *	0.44	0.15	3.04	0.004
	Variant	−0.05	0.20	−0.25	0.804
	Age	0.04	0.14	0.28	0.782
	EDSS	0.30	0.18	1.67	0.103
mBP *R* = 0.49; *R*^2^ = 0.24 F(4.42) = 3.33; *p* < 0.018	Sex *	0.37	0.16	2.35	0.023
	Variant	−0.02	0.22	−0.07	0.944
	Age	−0.05	0.16	−0.34	0.736
	EDSS	0.25	0.19	1.27	0.210
LFnu-RRI *R* = 0.48; *R*^2^ = 0.23 F(4.42) = 3.18; *p* < 0.022	Sex	0.14	0.16	0.88	0.384
	Variant	0.43	0.22	1.93	0.061
	Age	0.22	0.16	1.37	0.179
	EDSS	−0.30	0.19	−1.57	0.125
HFnu-RRI *R* = 0.47; *R*^2^ = 0.22 F(4.42) = 2.97; *p* < 0.029	Gender	−0.13	0.16	−0.79	0.432
	Variant	−0.42	0.22	−1.91	0.063
	Age	−0.21	0.16	−1.30	0.202
	EDSS	0.30	0.20	1.51	0.138
LF/HF-RRI *R* = 0.49; *R*^2^ = 0.24 F(4.42) = 3.36; *p* < 0.017	Sex	0.15	0.16	0.93	0.356
	Variant *	0.48	0.22	2.20	0.033
	Age	0.08	0.16	0.52	0.607
	EDSS	−0.21	0.19	−1.08	0.286
LF/HF *R* = 0.38; *R*^2^ = 0.15 F(4.42) = 6.1096; *p* < 0.143	Gender	0.11	0.17	0.65	0.519
	Variant	0.38	0.23	1.63	0.110
	Age	−0.05	0.17	−0.27	0.786
	EDSS	−0.07	0.21	−0.34	0.738
LF/HF-dBP *R* = 0.40; *R*^2^ = 0.16 F(1.53) = 10.23; *p* < 0.01	Sex	−0.26	0.15	−1.70	0.097
	Variant	0.25	0.21	1.18	0.245
	Age	0.25	0.15	1.62	0.113
	EDSS	0.20	0.19	1.06	0.296
HF-RRI *R* = 0.43; *R*^2^ = 0.19 F(1.53) = 2.4; *p* < 0.064	Sex	0.08	0.16	0.50	0.623
	Variant	−0.33	0.23	−1.46	0.152
	Age *	−0.34	0.16	−2.10	0.041
	EDSS	0.28	0.20	1.38	0.174

HR, heart rate; dBP, diastolic blood pressure; mBP, mean blood pressure; LFnu-RRI, low-frequency R-R interval in normalized units; HFnu-RRI, high frequency R-R interval in normalized units; LF/HF, ratio between low and high band for heart rate and blood pressure variability; LF/HF-RRI, ratio between low and high band for heart rate variability; LF/HF-dBP, ratio between low and high band for diastolic blood pressure variability; HF-RRI, high-frequency R-R interval; BETA (β), standardized beta coefficient; *t*-values; SE, standard error; R, squared; Statistically significant differences are indicated with * *p* < 0.05.

## Appendix A

**Table A1:** Spearman coefficients of correlation in MS subjects between baseline cardiovascular and autonomic parameters, age, disease duration and Expanded Disability Status Scale (EDSS) score.

	Age	EDSS	Disease Duration	HR	sBP	dBP	mBP	Delta HR	Delta sBP	Delta dBP	Delta mBP
Age (years)	1.00	0.46 **	0.44 **	0.00	0.04	0.29 *	0.19	−0.35 *	−0.04	−0.26	−0.13
HR (1/min)	0.00	0.37 **	0.04	1.00	0.12	0.21	0.17	−0.04	−0.18	−0.20	−0.23
sBP (mmHg)	0.04	0.23	−0.18	0.12	1.00	0.83 **	0.93 ***	−0.15	−0.13	0.02	−0.12
dBP (mmHg)	0.29 *	0.47 **	0.05	0.21	0.83 ***	1.00	0.96 **	−0.27	−0.15	−0.13	−0.18
mBP (mmHg)	0.19	0.38 **	−0.05	0.17	0.93 ***	0.96 ***	1.00	−0.23	−0.14	−0.08	−0.14
LFnu-RRI (ms^2^)	0.24	0.16	0.24	0.37 **	0.15	0.19	0.17	−0.15	0.03	0.09	0.12
HFnu-RRI (ms^2^)	−0.23	−0.16	−0.24	−0.36 *	−0.14	−0.19	−0.17	0.14	−0.03	−0.10	−0.13
LF-RRI (ms^2^)	−0.32 *	−0.25	−0.07	−0.54 ***	0.01	−0.17	−0.11	0.15	0.14	0.32 *	0.22
HF-RRI (ms^2^)	−0.34 *	−0.25	−0.18	−0.58 ***	−0.04	−0.22	−0.16	0.19	0.03	0.13	0.03
PSD-RRI (ms^2^)	−0.30 *	−0.22	−0.08	−0.59 ***	0.02	−0.15	−0.08	0.12	0.09	0.22	0.16
LF/HF-RRI [1]	0.25	0.18	0.26	0.39 **	0.13	0.19	0.17	−0.13	−0.01	0.05	0.08
LF/HF [1]	0.02	0.16	0.20	0.40 **	0.14	0.18	0.17	0.01	−0.07	0.05	0.03
LFnu-dBP (%)	−0.25	0.02	0.05	0.22	0.12	0.09	0.13	0.17	−0.22	−0.09	−0.13
HFnu-dBP (%)	−0.53 ***	−0.34 *	−0.18	−0.14	0.13	−0.04	0.03	0.15	0.02	0.12	0.01
LF-dBP (%)	−0.13	−0.12	−0.14	−0.21	0.17	0.06	0.14	0.17	0.11	0.25	0.21
HF-dBP (%)	−0.36 *	−0.34 *	−0.24	−0.31 *	0.16	−0.05	0.05	0.19	0.26	0.40 **	0.30 *
PSD-dBP (mmHg^2^)	−0.05	−0.17	−0.15	−0.30 *	0.10	0.00	0.07	0.12	0.25	0.35 *	0.32 *
LF/HF-dBP	0.31 *	0.30 *	0.21	0.19	−0.10	0.06	0.02	0.02	−0.14	−0.16	−0.06
LF/HF	0.02	0.16	0.20	0.40 **	0.14	0.18	0.17	0.01	−0.07	0.05	0.03
LFnu-sBP (%)	0.13	0.15	0.11	0.23	0.13	0.23	0.22	−0.01	−0.22	−0.18	−0.12
HFnu-sBP (%)	−0.17	−0.30 *	−0.19	−0.04	−0.05	−0.09	−0.10	0.03	−0.10	−0.07	−0.15
LF-sBP (%)	0.15	0.01	0.01	−0.12	0.17	0.13	0.18	0.01	0.11	0.18	0.16
HF-sBP (%)	−0.05	−0.20	−0.11	−0.23	0.07	−0.02	0.01	0.06	0.09	0.16	0.06
PSD-sBP (mmHg^2^)	0.07	−0.09	−0.03	−0.24	0.08	−0.03	0.05	0.04	0.26	0.29	0.24
LF/HF-sBP [1]	0.19	0.31 *	0.20	0.15	0.11	0.19	0.19	−0.02	−0.05	−0.02	0.05
LF/HF [1]	0.22	0.21	0.22	0.41 **	0.16	0.27	0.24	−0.09	−0.08	−0.01	0.03
BRS (ms/mmHg)	−0.33 *	−0.21	−0.08	−0.28	−0.35 *	−040 **	−0.39 **	0.13	−0.04	−0.01	−0.02
Total BEI (%)	−0.07	0.05	−0.16	−0.16	0.11	0.08	0.09	0.01	−0.11	−005	−0.10

Expanded Disability Status Scale (EDSS); HR, heart rate; sBP, systolic blood pressure; dBP, diastolic blood pressure; mBP, mean blood pressure; delta (change baseline-tilt); delta HR, delta heart rate; delta sBP, delta systolic blood pressure; delta dBP, delta diastolic blood pressure; delta mBP, delta mean blood pressure; LFnu-RRI, low-frequency R-R interval in normalized units; HFnu-RRI, high frequency R-R interval in normalized units; LF-RRI, low-frequency R-R interval; HF-RRI, high-frequency R-R interval; PSD-RRI, power spectral density R-R interval; LF/HF, ratio between low and high band for heart rate and blood pressure variability; LF/HF-RRI, ratio between low and high band for heart rate variability; LFnu-dBP, low frequency of diastolic blood pressure variability in normalized units; HFnu-dBP, high frequency of diastolic blood pressure variability in normalized units; LF-dBP, low frequency of diastolic blood pressure variability; HF-dBP, high frequency of diastolic blood pressure variability; PSD-dBP, power spectral density of diastolic blood pressure variability; LF/HF-dBP, ratio between low and high band for diastolic blood pressure variability; LFnu-sBP, low frequency of systolic blood pressure variability in normalized units; HFnu-dBP, high frequency of systolic blood pressure variability in normalized units; LF-sBP, low frequency of systolic blood pressure variability; HF-sBP, high frequency of systolic blood pressure variability; PSD-sBP, power spectral density of systolic blood pressure variability; LF/HF-sBP, ratio between low and high band for systolic blood pressure variability; BRS, baroreflex sensitivity; total BEI, baroreflex effectiveness; nu, normalized values; statistically significant differences are indicated with * *p* < 0.05, ** *p* < 0.01, *** *p* < 0.001.

**Table A2:** Spearman coefficients of correlation in MS subjects between post-tilt cardiovascular and autonomic parameters, age, disease duration and EDSS score.

	Age	EDSS	Disease Duration	HR	sBP	dBP	mBP	Delta HR	Delta sBP	Delta dBP	Delta mBP
Delta HR (1/min)	−0.35 *	−0.15	−0.26	−0.04	−0.15	−0.27	−0.23	1.00	0.08	0.19	0.07
Delta sBP (mmHg)	−0.04	−0.07	−0.03	−0.18	−0.13	−0.15	−0.14	0.08	1.00	0.84 ***	0.90 ***
Delta dBP (mmHg)	−0.26	−0.20	−0.13	−0.20	0.02	−0.13	−0.08	0.19	0.84 ***	1.00	0.89 ***
Delta mBP (mmHg)	−0.13	−0.13	0.01	−0.23	−0.12	−0.18	−0.14	0.07	0.90 ***	0.89 ***	1.00
Delta LFnu-RRI (ms^2^)	−0.45 ***	−0.22	−0.17	−0.14	0.08	−0.08	−0.01	0.41 **	−0.04	0.04	−0.09
Delta HFnu-RRI (ms^2^)	0.45 **	0.22	0.17	0.14	−0.08	0.08	0.01	−0.41 **	0.04	−0.04	0.09
Delta LF-RRI (ms^2^)	0.07	0.12	−0.04	0.46 ***	−0.04	0.03	0.03	−0.08	0.11	−0.08	−0.05
Delta HF-RRI (ms^2^)	0.38 *	0.26	0.20	0.45 ***	−0.08	0.10	0.04	−0.31 *	0.10	−0.07	0.06
Delta PSD-RRI (ms^2^)	0.20	0.16	0.09	0.52 ***	−0.07	0.04	0.00	−0.19	0.07	−0.07	−0.05
Delta LF/HF-RRI [1]	−0.32 *	−0.12	−0.03	0.11	0.16	−0.02	0.05	0.34 *	−0.07	0.08	−0.06
Delta LF/HF [1]	−0.31 *	−0.13	−0.08	0.18	0.16	−0.04	0.05	0.39 **	−0.07	0.13	−0.04
Delta LFnu-dBP (%)	−0.23	−0.11	−0.08	0.11	−0.09	−0.20	−0.13	0.30 *	0.11	0.18	0.16
Delta HFnu-dBP (%)	0.31 *	0.23	0.10	0.26	−0.21	−0.11	−0.15	0.07	−0.09	−0.20	−0.16
Delta LF-dBP (%)	−0.10	−0.03	0.05	0.07	0.21	0.04	0.12	−0.14	0.12	0.18	0.22
Delta HF-dBP (%)	0.25	0.34 *	0.10	0.35 *	0.08	0.10	0.11	−0.18	−0.10	−0.17	−0.10
PSD-dBP (mmHg^2^)	0.03	0.22	0.17	0.21	0.22	0.21	0.21	−0.35 *	−0.09	−0.07	−0.05
Delta LF/HF-dBP	−0.23	−0.22	0.01	−0.19	0.12	0.02	0.07	−0.04	0.13	0.29 **	0.26
Delta LF/HF	−0.31 *	−0.13	−0.08	0.18	0.16	−0.04	0.05	0.39 **	−0.07	0.13	−0.04
Delta LFnu-sBP (%)	−0.32 *	−0.14	−0.17	0.07	0.13	−0.07	0.04	0.27	0.26	0.41 **	0.30 *
Delta HFnu-sBP (%)	0.12	0.17	−0.06	0.26	−0.15	−0.10	−0.15	0.31 *	−0.03	−0.04	−0.09
Delta LF-sBP (%)	−0.15	−0.12	−0.10	0.00	0.16	−0.04	0.06	−0.03	0.21	0.26	0.25
Delta HF-sBP (%)	0.18	0.23	0.04	0.25	0.04	0.04	0.04	0.04	−0.07	−0.10	−0.06
Delta PSD-sBP (mmHg^2^)	−0.05	0.08	0.05	0.18	0.14	0.13	0.13	−0.25	−0.13	−0.12	−0.05
Delta LF/HF-sBP [1]	−0.25	−0.30 *	−0.08	−0.20	0.09	−0.05	0.03	−0.09	0.13	0.21	0.20
Delta LF/HF [1]	−0.27	−0.12	−0.06	0.15	0.20	−0.01	0.09	0.33 *	−0.02	0.16	−0.01

Expanded Disability Status Scale (EDSS); HR, heart rate; sBP, systolic blood pressure; dBP, diastolic blood pressure; mBP, mean blood pressure; delta, change baseline-tilt delta; delta HR, delta heart rate; delta sBP, delta systolic blood pressure; delta dBP, delta diastolic blood pressure; delta mBP, delta mean blood pressure; delta LFnu-RRI, delta low-frequency R-R interval in normalized units; delta HFnu-RRI, delta high frequency R-R interval in normalized units; delta LF-RRI, delta low-frequency R-R interval; delta HF-RRI, delta high-frequency R-R interval; delta PSD-RRI, delta power spectral density R-R interval; delta LF/HF, delta ratio between low and high band for heart rate and blood pressure variability; delta LF/HF-RRI, delta ratio between low and high band for heart rate variability; delta LFnu-dBP, delta low frequency of diastolic blood pressure variability in normalized units; delta HFnu-dBP, delta high frequency of diastolic blood pressure variability in normalized units; delta LF-dBP, delta low frequency of diastolic blood pressure variability; delta HF-dBP, delta high frequency of diastolic blood pressure variability; delta PSD-dBP, delta power spectral density of diastolic blood pressure variability; LF/HF-dBP, ratio between low and high band for diastolic blood pressure variability; delta LFnu-sBP, delta low frequency of systolic blood pressure variability in normalized units; delta HFnu-dBP, delta high frequency of systolic blood pressure variability in normalized units; delta LF-sBP, delta low frequency of systolic blood pressure variability; delta HF-sBP, delta high frequency of systolic blood pressure variability; delta PSD-sBP, delta power spectral density of systolic blood pressure variability; delta LF/HF-sBP, delta ratio between low and high band for systolic blood pressure variability; delta BRS, delta baroreflex sensitivity; delta total BEI, delta baroreflex effectiveness; nu, normalized values; statistically significant differences are indicated with * *p* < 0.05, ** *p* < 0.01, *** *p* < 0.001.
